# A Thematic Analysis Exploring Bowel Cancer Screening Promotional Visuals in the United Kingdom and India

**DOI:** 10.3928/24748307-20260304-01

**Published:** 2026-04

**Authors:** Ioanna Yfantidou, Marek Palace, Christian von Wagner, Sandro Stoffel, Carlos Santos Barea, Lee Smith, Brandon May, Meghna Srivastava, Jazzine Samuel

**Affiliations:** a Liverpool Business School; b School of Psychology; c Liverpool John Moores University, Liverpool, Behavioural Science in Health, University College London, London, United Kingdom;; d Institute of Pharmaceutical Medicine (ECPM), and Health Economics Facility, Department of Public Health, University of Basel, Basel, Switzerland, and Department of Behavioural Science and Health, University of London, London, UK;; e School of Arts and Design, Liverpool John Moores University, Liverpool; f Centre for Health, Performance and Wellbeing, Anglia Ruskin University, Cambridge, UK and Department of Public Health, Faculty of Medicine, Biruni University, Istanbul, Turkey;; g School of Psychology, Florida Institute of Technology, Florida, United States;; h The Centre for Human Brain Health, University of Birmingham, Birmingham, United Kingdom.; i Behavioural Science & Early Diagnosis of Cancer, University College London, London, United Kingdom.

## Abstract

**Objective:**

The purpose of this qualitative study is to explore and identify elements used in bowel cancer screening visuals that promote health literacy and participation in screening tests in the United Kingdom and India.

**Methods:**

A total of 17 participants from the United Kingdom and India took part in a thematic analysis study. Individuals were eligible for this study, if they were within the National Health Service bowel cancer screening target between ages 50 and 74 years. A total of 58 bowel cancer screening promotional images were presented to the participants, and they had to indicate whether they believed the image effectively encouraged participation in bowel cancer screening. Then, participants were invited for a remote interview to explore their verbal constructions of the bowel cancer screening images. The interview questions were designed to explore participants' preferences and aversions toward the visual elements used in promoting health literacy around bowel cancer screening.

**Key Results:**

The findings highlight key visual features that resonate with the target audience and those that may hinder the effectiveness of screening messages. Encouraging elements include images of happy families, the portrayal of screening consequences, clear details about the screening process, and the inclusion of health care professionals. The study also underscores the importance of using both gain-framed and loss-framed messages. In contrast, discouraging elements, such as ambiguous images, such as pictures of toilets, and visuals evoking negative emotions, such as pictures showing internal organs, were identified as barriers to engagement. These visuals may confuse audiences or evoke feelings of disgust, which can deter individuals from participating in screening. The findings suggest that screening promotion materials should incorporate clear, informative visuals that directly relate to the screening process, such as test kits and health care professionals.

**Conclusions:**

The study provides practical recommendations for designing effective, culturally relevant promotional materials that balance informational content with emotional appeal, improving the likelihood of increasing bowel cancer screening uptake.

## Abstract (plain language summary)

**Plain Language Summary: **This study looked at how pictures used to promote bowel cancer screening are understood by people age 50 to 74 years in the UK and India. Participants viewed 58 images and discussed which ones encouraged them to take part in screening. They preferred visuals showing families, health professionals, clear test information, and the benefits or consequences of screening. Images that were confusing or unpleasant are found to put people off. The findings suggest that screening campaigns should use simple, relatable, and informative visuals to help more people feel confident about taking the test.

Bowel cancer or colorectal cancer accounts for 10 per cent of all cancer deaths in the United Kingdom (Cancer Research UK, n.d.) and is the fourth leading cause of cancer deaths worldwide (Ferlay et al., 2015). Early detection plays a crucial role in the successful treatment and reduction of mortality associated with this type of cancer (Levin et al., 2018). One effective screening method is the Fecal Immunochemical Test (FIT), which detects the presence of blood in the fecal stool, a potential indicator of bowel cancer (Heer et al., 2023). By identifying the disease in its early stages through FIT screening, health care professionals can provide timely interventions, leading to improved patient outcomes and a decrease in the overall mortality rate attributed to bowel cancer (Zheng et al., 2023).

Organized screening programs are widely prevalent in most parts of the world (Navarro et al., 2017), which have often used visual imagery to promote uptake and participation in the screening procedure. Despite these programs and promotional efforts, there is a large disparity in uptake of screening in these programs with some programs having a completion rate as low as 16% in some countries (Navarro et al., 2017).

## The Role of Visual Images in Health Communication

Previous research on increasing cancer screening health literacy highlights the need for social media-based communication methods (Lee et al., 2021). Visual media, such as photographs, videos, pictograms, and infographics, are widely employed and acknowledged as highly effective components in the field of health communication campaigns and initiatives (Barros et al., 2014). Images play a crucial role in social education by equipping people with the skills to learn and critically analyze the world around them (Kellner & Share, 2019).

Theoretical frameworks concerning visual persuasion shape our understanding of visual perception. The theory of planned behavior (He, 2023) highlights the effectiveness of demographic factors like age, origin and income, which influence the success of visual persuasion and Pierce's Visual Semiotic System (Chong et al., 2023) which establishes a triadic relationship between signs, objects, and interpretants. This model emphasizes how visual elements function as vehicles of spatial meaning and how they contribute to the creation of meaning through a dynamic semiotic process. Peirce's concept of hypoicons emphasizes how visual signs acquire meaning through cultural habits and collateral experience, which is relevant to our study as in the UK a symbolic image like the red ribbon, may evoke cancer awareness, whereas in India the same symbol may not carry the same connotation. In addition, the visual of a screening kit (Pierce's sign) in some advertisements may be linked to the concept of early detection (Pierce's object), but the interpretant depends on the viewer's prior knowledge and cultural context. Together, these frameworks establish that images possess unique semantic and syntactic properties that make them more effective message carriers than text alone.

The use of visual imagery has shown to impact adherence to health instructions, especially in patients with lower literacy levels (Houts et al., 2006). There have been many examples of graphic content being successfully used to promote change in health behavior and spread awareness (King, 2017). The inclusion of images in health risk communication can significantly influence how messages are received, particularly across diverse cultural backgrounds and demographic groups (Manno et al., 2018). Visuals may interact with factors such as cognitive processing needs, cultural symbolism, and lived experiences, thereby shaping the viewer's understanding and engagement with the message (Manno et al., 2018). Existing research evaluating the contents of promotional materials in print media focus mainly on the textual components of the images (Jensen, 2012). These make suggestions on optimizing visual components that are broad, vague and lack actionable points of improvement based on empirical research (King, 2015). Studies using physiological metrics (e.g., eye tracking) has revealed that health promotion advertisements, with visual elements captured, were viewed more times and for longer compared to advertisements with just text elements (Milošević et al., 2024) implying that the visual elements work better at capturing and sustaining the attention of the viewer. Although the effectiveness of images in health promotion is well established, selecting specific visual elements should be guided by evidence on how the emotional responses they evoke may influence health-related decision-making (Santos et al., 2022).

## Visual Images in Bowel Cancer Screening Promotion

Studies exploring the effect of media in promotion of health behavior, especially cancer screening uptake (Jedele & Ismail, 2010), has found that there is a positive relationship between screening uptake behavior and exposure to demographically targeted visual campaigns promoting cancer screening such as billboards. Development of visual and media tools targeted to specific communities have shown exemplary results in acceptability and positive intentions in screening (Kruse-Diehr et al., 2024). For instance, a recent study revealed that current visuals used in bowel cancer screening promotion, with images focused on the technical aspects of the screening process, rarely engaged with the audience on an emotional level. The findings also highlighted the need for more emotionally impactful and narrative-driven visuals (Gatting, 2022). In contrast, didactic videos positively influenced screening intentions only among individuals who had received an invitation letter but did not undergo screening, and those who had not yet received an invitation (Scaglioni et al., 2024), highlighting a further call for tailored promotional strategies to be developed for specific groups.

## Aim of the Current Study

Despite the widespread use of images in promoting health behaviors, previous research has not thoroughly investigated the impact of different visual elements on bowel cancer screening intentions. Moreover, there is a lack of understanding regarding how people perceive different aspects of these images, and which specific elements are most effective in encouraging positive health behaviors or potentially have an adverse effect on such behaviors. Our current study aims to explore perceptions and attitudes toward existing bowel cancer screening images by using binary ratings and semi-structured interviews to identify which elements of these images are effective or ineffective. Additionally, the study aims to shortlist a selection of 58 images for a future quantitative and physiological study.

## Methods

### Participants

A total of 17 participants took part in this qualitative study (*N* = 9 [female]), *N* = 8 [male]). Individuals were recruited from the UK (*N* = 8) and India (*N* = 9) through a mix of opportunistic sampling and purposive sampling. Recruited participants ranged in age from 50 to 68 years (*M* = 57 years, *SD* = 5.5). Opportunistic sampling allowed participants to be recruited at a fast pace, while purposive sampling ensured a diversity of factors which may have influenced perceptions on bowel cancer screening. These included ethnicity, socio-economic status and highest level of education (Campbell et al., 2020; Guiriguet et al., 2017). Individuals were eligible for this study, if they were within the National Health Service (NHS) bowel cancer screening target ages of 50 to 74 years old (NHS, 2024) and had not completed a university-level education, since lower educational levels was found to be associated with a decreased probability of participating in cancer screening (Simpson et al., 2020; Willems & Bracke, 2018). According to the latest Office for National Statistics (2021) data, 33.6% of UK population is university-educated. Yet, Census does not provide a breakdown by age and it can be assumed that the percentage for people older than age 50 years should be much lower as older generations historically had less access to higher education compared to younger cohorts. Individuals were provided with a Participant Information Sheet and written informed consent was obtained before commencing. Ethical approval for the study has been granted by the university's Ethics Committee.

### Collection of Stimuli (Existing Bowel Cancer Screening Promotion Images)

A total of 250 images including posters, visual aids, and promotional materials sourced from national screening and health promotion campaigns from all over the world. The collected images aimed at increasing awareness and promoted cancer screening. The methods used to find the relevant images included using image search across various internet sources (Google, Bing, Opera). A combination of terms was used to find images pertaining to cancer screening promotion. Specifically, the search process involved employing relevant keywords such as “cancer screening promotion images,” “cancer screening promotion posters,” and “bowel cancer screening promotion images.”

The collected images originated from diverse sources, including private entities, public health organizations and government programs. These sources spanned over 20 countries and organizations including the UK, Japan, Hong Kong, South Korea, USA, Canada, Spain, Germany, South Korea, Mexico, Portugal, Brazil, Australia, New Zealand, Thailand, Ireland, India, Indonesia, Malaysia, Singapore, Philippines, Saudi Arabia, Qatar, UAE, Lebanon, Bahrain, France, Pakistan, Angola, Cape Verde, Trinidad and Tobago, Honduras, along with pan-European and American organizations.

Following the initial pool of the images, a filtering process was undertaken to shortlist the images for further stages of the study. Images were examined to exclude those reliant on their accompanying text for comprehension, featuring inseparable text elements, and images that were too specific to other forms of cancer such as images with the pink ribbon symbolizing breast cancer, images with depictions of specific organs related to other forms of cancer such as lungs, prostate, breasts, and images featuring individuals not within the intended demographic range of age 50 years and older. Following this filtering, 58 images remained for subsequent analysis in this study.

### Creation of Images with Generic Text

The existing texts on these shortlisted images were removed using Adobe Firefly's Generative Fill feature. This tool used generative AI to erase the text and fill in the gaps, ensuring the edited areas blended perfectly with the rest of the image.

This process gave us images with just visuals in them and no text elements. We then added the generic text, “Bowel cancer screening saves lives” to these images. This was done to ensure that our study's assessment focused exclusively on the visual elements, without any influence from the text content. Having the same generic, standard text on every image eliminated any bias caused by different texts to allow any difference in assessment of the images by participants to be based on its visual elements. If the image featured human subjects, the text was added near the face and eyes of the humans as images with faces, people or animals may have caught the viewer's attention, therefore prompting the viewers to read the text (Banerjee et al., 2013; Sajjacholapunt & Ball, 2014). For participants in India, the generic text was translated into two additional regional languages, Bengali and Hindi, to accommodate the participants recruited through opportunistic sampling in India.

### Procedure

The study began with participants completing an online survey rating existing bowel cancer screening promotion images. The survey was conducted to finalize a number of bowel screening promotional images for a larger survey which will further explore perceptions on these shortlisted images through survey-based ratings and eye tracking tools to extract the most and least effective elements. After collecting basic demographics (age and gender), participants rated a total of 58 bowel cancer screening promotional images on whether they believed the image effectively encouraged participation in bowel cancer screening through a binary thumbs up and thumbs down mechanism. The thumbs up/thumbs down binary rating system is widely used in marketing research and digital platforms to measure user sentiment, particularly in contexts where quick feedback is needed. According to Appcues software business, this binary rating system reduces cognitive load, as it is easier for participants to respond quickly, and captures sentiment, which is useful for gauging general approval or disapproval of visuals, messages, or products (Newswire, 2023). **Table A** shows participants' reactions to the 58 bowel cancer screening images.

**Table A:**
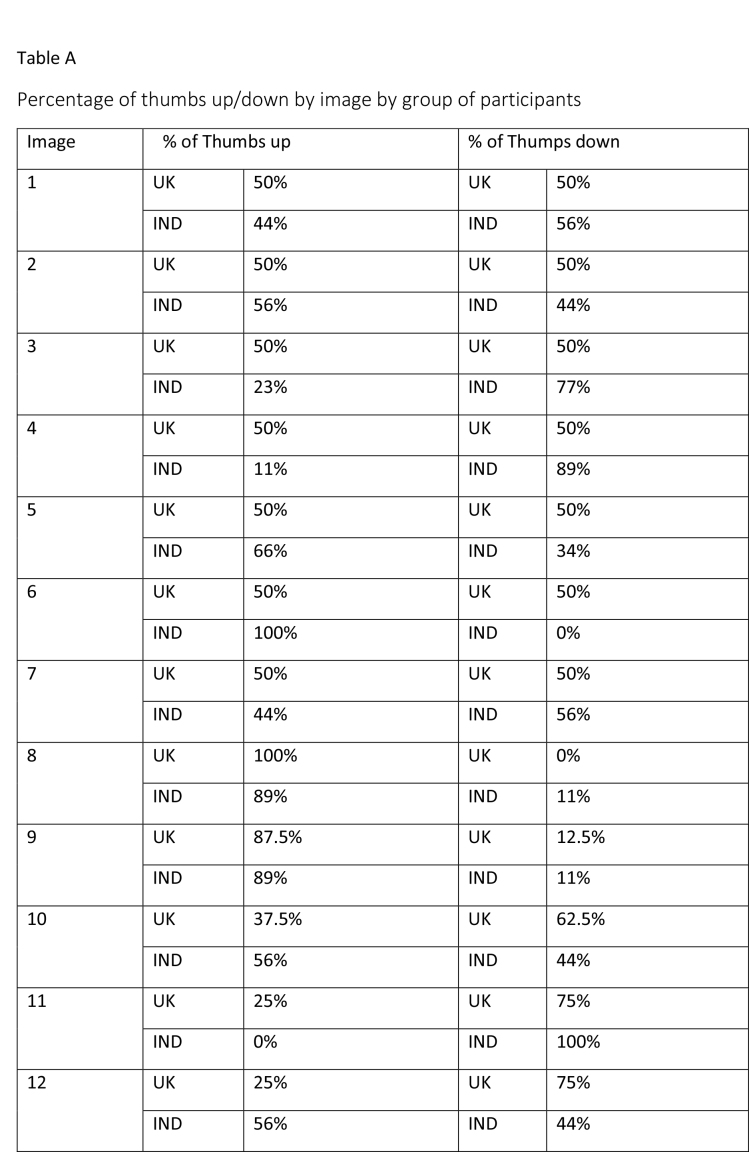
Percentage of thumbs up/down by image by group of participants

Image	% of Thumbs up		% of Thumps down	
1	UK	50%	UK	50%
	IND	44%	IND	56%
2	UK	50%	UK	50%
	IND	56%	IND	44%
3	UK	50%	UK	50%
	IND	23%	IND	77%
4	UK	50%	UK	50%
	IND	11%	IND	89%
5	UK	50%	UK	50%
	IND	66%	IND	34%
6	UK	50%	UK	50%
	IND	100%	IND	0%
7	UK	50%	UK	50%
	IND	44%	IND	56%
8	UK	100%	UK	0%
	IND	89%	IND	11%
9	UK	87.5%	UK	12.5%
	IND	89%	IND	11%
10	UK	37.5%	UK	62.5%
	IND	56%	IND	44%
11	UK	25%	UK	75%
	IND	0%	IND	100%
12	UK	25%	UK	75%
	IND	56%	IND	44%

**Table:**
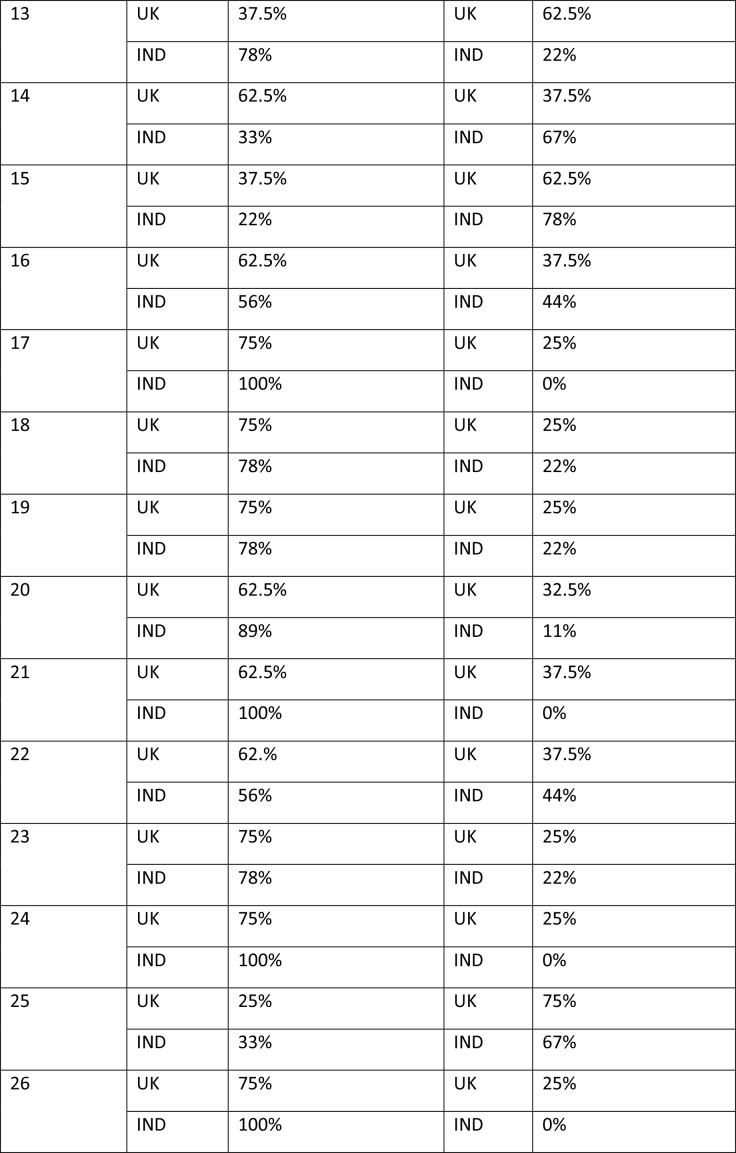


13	UK	37.5%	UK	62.5%
	IND	78%	IND	22%
14	UK	62.5%	UK	37.5%
	IND	33%	IND	67%
15	UK	37.5%	UK	62.5%
	IND	22%	IND	78%
16	UK	62.5%	UK	37.5%
	IND	56%	IND	44%
17	UK	75%	UK	25%
	IND	100%	IND	0%
18	UK	75%	UK	25%
	IND	78%	IND	22%
19	UK	75%	UK	25%
	IND	78%	IND	22%
20	UK	62.5%	UK	32.5%
	IND	89%	IND	11%
21	UK	62.5%	UK	37.5%
	IND	100%	IND	0%
22	UK	62.%	UK	37.5%
	IND	56%	IND	44%
23	UK	75%	UK	25%
	IND	78%	IND	22%
24	UK	75%	UK	25%
	IND	100%	IND	0%
25	UK	25%	UK	75%
	IND	33%	IND	67%
26	UK	75%	UK	25%
	IND	100%	IND	0%

**Table:**
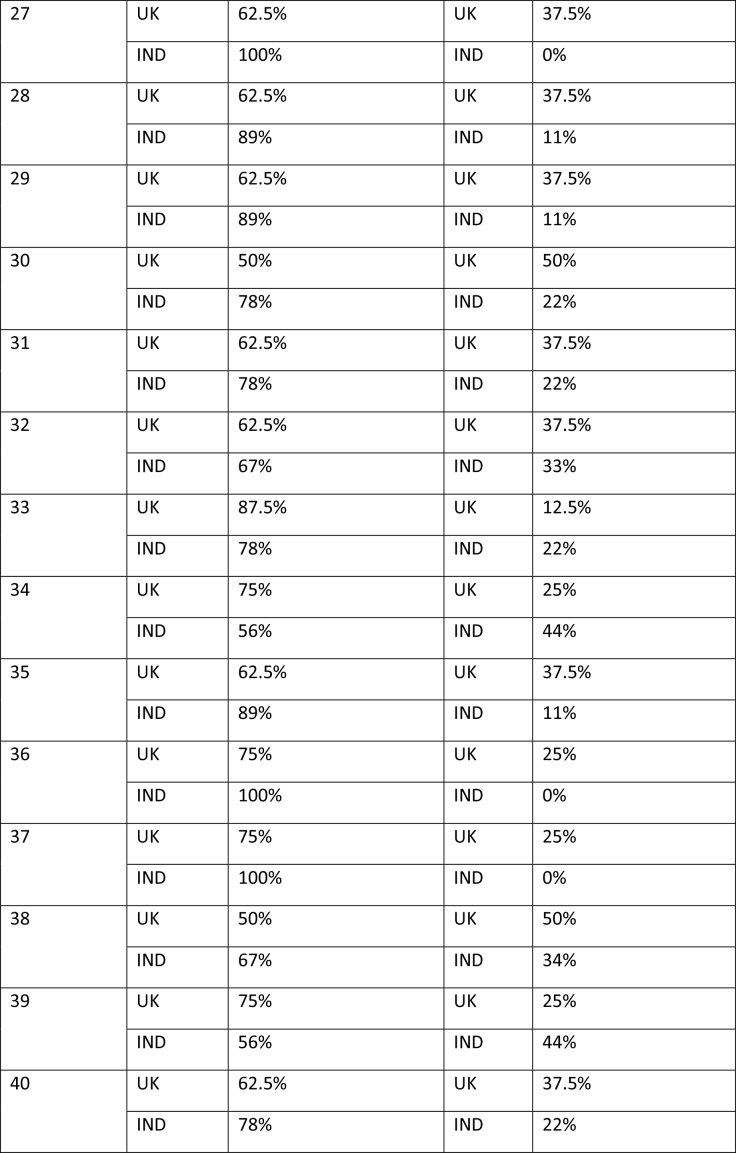


27	UK	62.5%	UK	37.5%
	IND	100%	IND	0%
28	UK	62.5%	UK	37.5%
	IND	89%	IND	11%
29	UK	62.5%	UK	37.5%
	IND	89%	IND	11%
30	UK	50%	UK	50%
	IND	78%	IND	22%
31	UK	62.5%	UK	37.5%
	IND	78%	IND	22%
32	UK	62.5%	UK	37.5%
	IND	67%	IND	33%
33	UK	87.5%	UK	12.5%
	IND	78%	IND	22%
34	UK	75%	UK	25%
	IND	56%	IND	44%
35	UK	62.5%	UK	37.5%
	IND	89%	IND	11%
36	UK	75%	UK	25%
	IND	100%	IND	0%
37	UK	75%	UK	25%
	IND	100%	IND	0%
38	UK	50%	UK	50%
	IND	67%	IND	34%
39	UK	75%	UK	25%
	IND	56%	IND	44%
40	UK	62.5%	UK	37.5%
	IND	78%	IND	22%

**Table:**
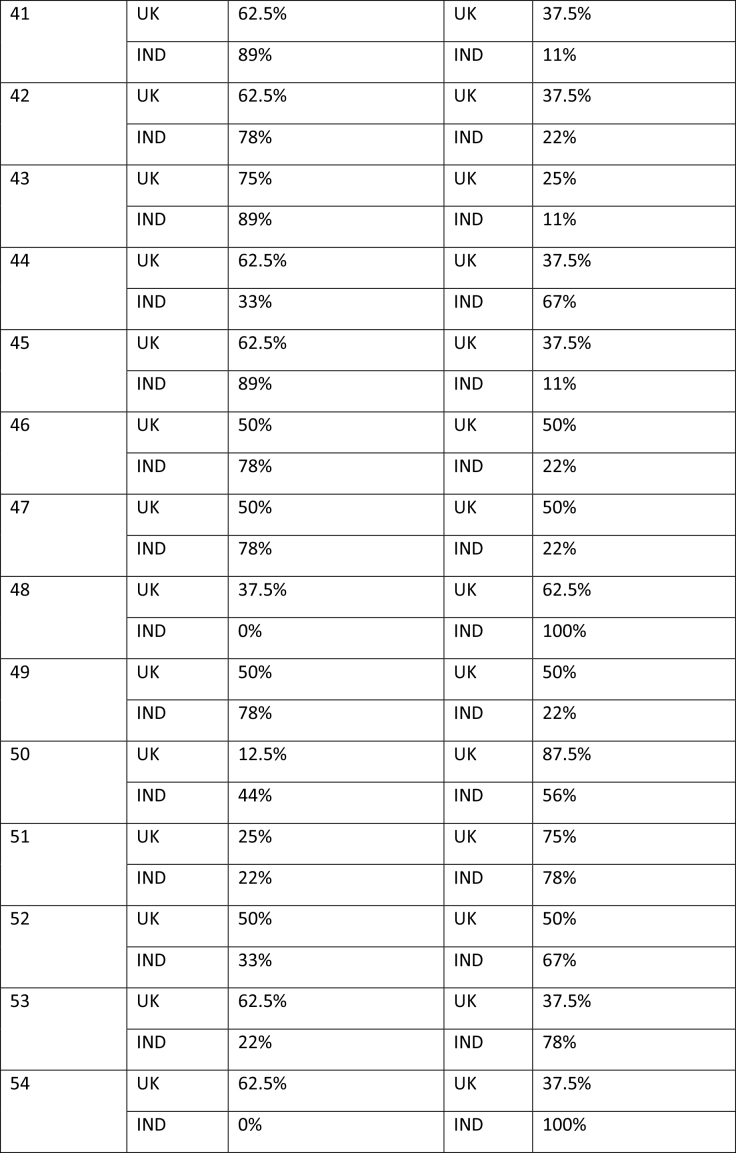


41	UK	62.5%	UK	37.5%
	IND	89%	IND	11%
42	UK	62.5%	UK	37.5%
	IND	78%	IND	22%
43	UK	75%	UK	25%
	IND	89%	IND	11%
44	UK	62.5%	UK	37.5%
	IND	33%	IND	67%
45	UK	62.5%	UK	37.5%
	IND	89%	IND	11%
46	UK	50%	UK	50%
	IND	78%	IND	22%
47	UK	50%	UK	50%
	IND	78%	IND	22%
48	UK	37.5%	UK	62.5%
	IND	0%	IND	100%
49	UK	50%	UK	50%
	IND	78%	IND	22%
50	UK	12.5%	UK	87.5%
	IND	44%	IND	56%
51	UK	25%	UK	75%
	IND	22%	IND	78%
52	UK	50%	UK	50%
	IND	33%	IND	67%
53	UK	62.5%	UK	37.5%
	IND	22%	IND	78%
54	UK	62.5%	UK	37.5%
	IND	0%	IND	100%

**Table:**
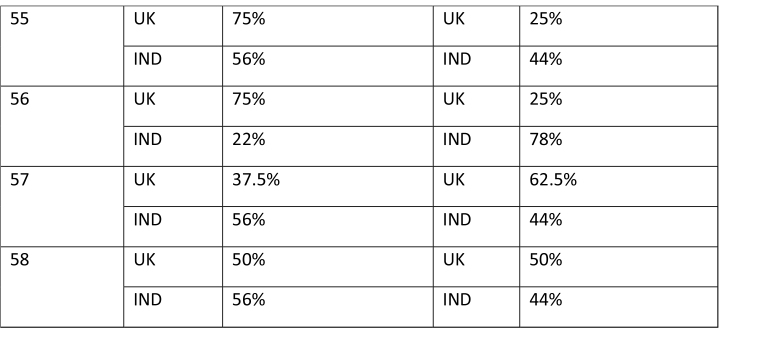


55	UK	75%	UK	25%
	IND	56%	IND	44%
56	UK	75%	UK	25%
	IND	22%	IND	78%
57	UK	37.5%	UK	62.5%
	IND	56%	IND	44%
58	UK	50%	UK	50%
	IND	56%	IND	44%

Immediately following completion of the image rating survey, participants were invited for a remote interview to explore their perceptions on the bowel cancer screening images. Participants relied on memory but were encouraged to ask the researcher to show them a specific image again if they deemed necessary. Interviews were conducted between January 2024 to March 2024, each lasting approximately 12 to 16 minutes, with an average of 6 minutes. Two researchers conducted the interviews, with R1 conducting the interviews with UK participants, while R2 conducted interviews with Indian participants. All interviews were audio-recorded and transcribed with Microsoft Teams and Otter AI, an AI based meeting assistant that automatically transcribed audio to text (Otter.ai, 2024). Due to difficulties recruiting Indian participants that were fluent in English, six interviews were conducted in native Indian languages (3 in Hindi, 3 in Bengali) before their transcripts were translated into English. While every effort was made to ensure accurate and faithful translation, we acknowledge the potential for meaning loss or shifts in nuance during this process. To mitigate this, translations were reviewed by bilingual researchers familiar with both the linguistic and cultural contexts of the participants.

### Interview Schedule

A semi-structured interview schedule was developed to explore participants' attitudes towards the bowel cancer screening images they viewed in the image rating survey. The schedule was designed to qualitatively capture the participants' perceptions, attitudes and emotional responses during the interview. The interview questions were designed to elicit the participants' overall impressions of the bowel cancer screening promotion images, as well as to identify specific aspects of the images that they found appealing or unappealing. The questions aimed to uncover the participants' preferences and dislikes regarding the visual elements used to promote bowel cancer screening. Topics included what elements respondents thought should be added or removed from the survey image, if there was a certain type of image, they wish to have seen that wasn't present in the survey of images, what they perceived as an “ideal” image to promote bowel cancer screening images, and potential cultural elements that should be added or avoided in bowel cancer screening images.

### Analysis

Thematic analysis is used in this study, as it is an effective tool for capturing the nuances of a phenomena that quantitative methods may not fully capture (Terry et al., 2017). The aim is to explore patterns and themes within textual data, as such, the team followed an inductive process that allowed themes to emerge organically from the data. This ensured findings were grounded in participants' lived experiences. Both R1 and R2 analyzed the data following Richards and Hemphill's (2018) 6-step method of collocative qualitative analysis. Following this, the researchers coded the transcripts independently whilst holding three separate meetings to discuss the coding and continually update the coding manual to reflect ongoing analysis. Initial coding was conducted separately, followed by a comparison of codes to assess inter-rater agreement. The level of agreement was calculated using Cohen's Kappa (0.68), resulting in an agreement rate of 85%, indicating substantial agreement. Discrepancies in coding were resolved through discussion and consensus-building. This process ensured that the final coding framework was both rigorous and reflective of the data. The use of multiple coders and a structured resolution process helped to minimize bias and enhance the credibility and trustworthiness of the findings.

## Results

The first few questions of the interview are related to whether participants had heard of bowel cancer, have had a bowel cancer screening and consider themselves as a high-risk individual. Interestingly, only one participant from India had not heard of bowel cancer (11%). Also, four participants from the UK (50%) had had a bowel cancer screening test and one of them considered themselves high-risk individual (13%). None of the participants from India had ever had a bowel cancer screening test.

### Themes

Two themes were developed during analysis: (1) Encouraging elements and (2) Discouraging elements. The encouraging elements theme had 3 subthemes (1) Portraying the consequences of screening decisions, (2) Images should provide enough detail about bowel cancer screening, and (3) Images with health care professionals are encouraging. These themes offer insight into how individuals from diverse cultural and demographic backgrounds perceive and respond to existing bowel cancer screening visuals. They help identify which visual elements may encourage or discourage participation, highlighting the importance of inclusive and culturally sensitive design in health communication. The themes are presented with illustrative quotes in the following section. Participants are referred to by their identification number and which country they were from to provide contextual understanding.

### Encouraging Elements

Participants identified specific elements within the presented images that they perceived as encouraging in promoting bowel cancer screening. This theme delved into the visual components that resonated with the participants and were deemed impactful in conveying the importance of undergoing screening for bowel cancer. Within this theme, the current study identified three subordinate themes. These subthemes, discussed in detail below, provide insights into specific factors participants identified as potentially increasing screening participation.

***Sub theme 1: Portraying the consequences of screening decisions.*** Images that conveyed either the positive consequences of screening or negative consequences of not screening were verbally constructed as important for conveying the seriousness of the message. Specifically, in terms of positive consequences, participants expressed their wish to see images that depicted individuals who had the test and screened clear for signs of bowel cancer.

“I think a positive result would be a good one. You know somebody that had got it done and then you, when they get the all clear I feel like that sort of thing.” (Participant 2, UK)

On the other hand, a few participants found that portraying only positive images did not highlight the seriousness of choosing not to take part. For example, one participant expressed that showing happy families leaves out the importance of what happens when someone chooses not to do the test.

“Like, yeah, put a happy couple, and happy family there, but it needs to have something there showing you what can happen if you don't do it.” (Participant 4, UK)

This same argument was expressed by Indian participants:

“I think people need to know when to go for screening, […] like if I see someone who ignored screening and later got cancer or had health difficulties I would be scared and would not want to be that person”

(Participant 6, India)

This is in line with the Value Function Prospect theory (Levy & Levy, 2002), which suggests that when individuals face behavioral choices that involve risk or uncertainty, such as deciding whether to undergo bowel cancer screening, they are more likely to take these risks when the information about their options is presented in terms of the relative disadvantages, losses, or costs associated with each choice (Dillard & Pfau, 2002).

Moreover, for participants in both the UK and India, images of happy families were generally well-received as they were believed to have positively depicted what disease-free life would be like if one participated in timely bowel cancer screening.

“Also, I liked the images of families because if screening can lead to early treatment of the disease, then the person will be able to lead a happy family life.” (Participant 1, India).

“I like the ones with people around in the open air. It showed they've got a healthy lifestyle.” (Participant 1, UK)

This is consistent with the finding that persuasion strategies, when framed to highlight the gains of certain behavior rather than the fear of loss, is more effective (Schneider et al., 2001).

***Subtheme 2: Images should provide enough detail about bowel cancer screening.*** This theme reflects participants' desire for images that provide a clear understanding of the bowel cancer screening process. The majority of participants (*n* = 11) mentioned that not enough information was given for them to gain an understanding of what screening involved and why it was important.

“There were a lot with happy families happy couples and that's not really relevant, it's the test and getting it done… It's alright saying someone's happy after the event. But what causes it? Why is it happening?” (Participant 4, UK)

This highlights the importance of ensuring that images used in bowel cancer screening promotion materials have a clear and direct connection to the topic (Gatting, 2022). Ambiguity in the visuals may lead to confusion and reduced effectiveness in conveying the intended message. Instead, further depictions of the test kit were encouraged to highlight the actual test and make the screening process clearer:

“[I like the ones with] the test itself and obviously they were sitting there with the tests with the leaflet and all that sort of stuff that's kind of kind of thing you were doing. But also the test itself.”

(Participant 6, UK)

“I think they're about 4 or 5 that didn't have the screening kit to like highlight what we're there for.”

(Participant 7, UK)

By including recognizable elements related to the screening process, such as the test kit, images may more effectively communicate their relevance to bowel cancer screening. This effect however was considered not efficient among participants in India where there is a lack of organized bowel cancer screening:

There were some images with a small thing [test kit] in their hands but I didn't know what that was.

(Participant 3, India)

To improve the content of screening promotional images and provide more information, one participant suggested providing a QR code that individuals can scan for more detailed information.

“Maybe if you want to have more information on it, they could have a website or a QR to press on, just get more information onto it as well.” (Participant 5, UK)

***Subtheme 3: Images with health care professionals are encouraging.*** Participants expressed that images depicting doctors, nurses, or other medical staff could help convey the importance and credibility of the screening message:

“Yes, [I liked] the doctor with a stethoscope around her neck telling you that bowel screening saves lives.”

(Participant 3, UK)

Similarly, a participant from India suggested:

“If the image shows a doctor explaining the screening process or encouraging people to get tested, it might be more convincing. Doctors are respected figures, and their advice carries weight.”

(Participant 8, India)

These findings align with evidence that physician endorsement was one of the strongest predictors of colorectal cancer screening uptake (Tsipa et al., 2021). Moreover, images of health care workers may help bridge knowledge gaps and provide reassurance about the screening procedure. By portraying the involvement of trained professionals, visuals could help alleviate fears or uncertainties that may deter individuals from participating in screening (Prowse et al., 2024).

Overall, these findings highlight the potential benefits of incorporating images of health care professionals into bowel cancer screening promotion materials. The trust and credibility associated with medical figures could enhance the persuasive impact of screening messages and provide a supportive framework for individuals considering participation. Despite the positive comments from participants both from the UK and India, it needs to be highlighted that more Indians than British expressed trust in medical figures, which illustrated that the finding is possibly culturally specific and might not apply among participants who have more distrust of health care professionals.

### Discouraging Elements

This theme reflects aspects of the images that participants felt discouraged them from participating in screening or made it difficult for them to understand the message that the images were trying to convey.

***Subtheme 1: Emotionally disruptive visuals.*** Images that included items with negative connotations, such as toilets and organs, were often perceived as off-putting by participants. These images elicited feelings of disgust and concerns about hygiene, which could potentially discourage individuals from engaging with the screening process. One participant expressed their discomfort with images of internal organs, stating,

“And the pictures of the guts as well, because it makes you feel sick to look at.”

(Participant 1, UK)

Similarly, the presence of toilets in images was seen as problematic by participants in India:

“I think the toilet must be avoided as far as it can be. It looks very icky and dirty”

(Participant 4, India)

This sentiment suggests that associating the screening process with toilets may reinforce negative stereotypes and create barriers to participation. Research has shown that emotions play a significant role in health decision-making, and negative emotions such as disgust can hinder engagement with health behaviors (Santos et al., 2022). A study by Reynolds et al. (2013) found that individuals with higher levels of disgust sensitivity were less likely to participate in colorectal cancer screening. This finding underscores the importance of avoiding visual elements that may trigger disgust or other negative emotions in health communication materials.

Corroborating this, another participant noted:

“I didn't like the picture of the intestines. It was just too graphic, too in your face. It made me feel a bit queasy looking at it.”

(Participant 7, India)

This finding supports previous research showing that images with graphic or disturbing content can elicit negative emotional reactions, which may subsequently influence health-related attitudes and behaviors. (Shi et al., 2023).

***Subtheme 2: The risk of gender bias in health messaging.*** Another concern raised by participants was the potential for images featuring only women to be misinterpreted, particularly by male viewers, as relating to other types of cancer when insufficient context was provided. One participant noted:

“For me personally it was just a woman on the picture. I would probably discard thinking is more related like for instance like a breast cancer scenario.”

(Participant 5)

This underscores the need for images to be inclusive and representative of the target audience for bowel cancer screening (Verma, 2022). By featuring a diverse range of individuals and providing clear visual cues connecting the image to bowel cancer, promotional materials may avoid unintended associations with other health topics.

Considering the themes and subthemes, as well as the binary image rating of the 58 images, **Table 1** shows what subthemes each of the images relates to, and the average positive or negative ranking per subtheme.

**Table 1:**
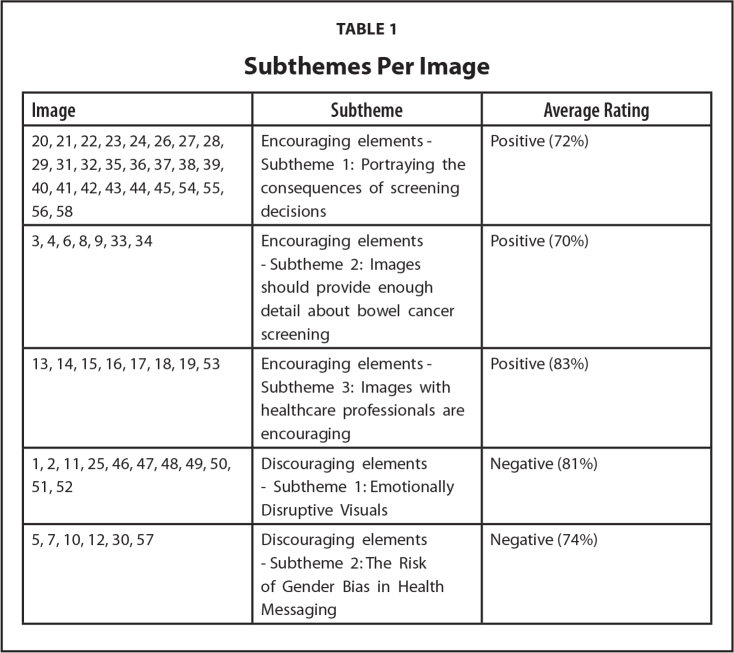
Subthemes Per Image

**Image**	**Subtheme**	**Average Rating**
20, 21, 22, 23, 24, 26, 27, 28, 29, 31, 32, 35, 36, 37, 38, 39, 40, 41, 42, 43, 44, 45, 54, 55, 56, 58	Encouraging elements - Subtheme 1: Portraying the consequences of screening decisions	Positive (72%)
3, 4, 6, 8, 9, 33, 34	Encouraging elements - Subtheme 2: Images should provide enough detail about bowel cancer screening	Positive (70%)
13, 14, 15, 16, 17, 18, 19, 53	Encouraging elements - Subtheme 3: Images with healthcare professionals are encouraging	Positive (83%)
1, 2, 11, 25, 46, 47, 48, 49, 50, 51, 52	Discouraging elements - Subtheme 1: Emotionally Disruptive Visuals	Negative (81%)
5, 7, 10, 12, 30, 57	Discouraging elements - Subtheme 2: The Risk of Gender Bias in Health Messaging	Negative (74%)

## Discussion

The current qualitative study aimed to explore the encouraging and discouraging elements of images used in bowel cancer screening promotion materials, as verbally constructed by participants from the United Kingdom and India. The findings provide valuable insights into the visual components that resonate with the target audience and those that may hinder the effectiveness of screening messages.

Participants identified several encouraging elements in the presented images, including the combination of depictions of organs and families, happy families, portraying the consequences of screening decisions, providing sufficient detail about the screening process, and the presence of health care professionals. These findings align with existing research on effective health communication strategies, including interventions aimed at facilitating adherence to treatment recommendations for those low in health literacy (Harley et al., 2023). Interestingly, regardless of the culture, the images featuring toilet elements (e.g., toilet roll, or toiler bowl) were rated lowest and the images featuring smiling couples were rated highest and were commented upon most favorably. This, in turn, corresponds to the cross-cultural aversion of images associated with the human stool, that are still used in bowel cancer screening promotion; and a clear preference for those depicting happy relationships, the positive visual motif of which is already used in businesses dealing with sensitive topics, such as life insurance companies (almost never portraying any funeral elements) around the world (Lehtonen, 2014; Pareek et al., 2022).

The expressed preference for images of happy families and health care professionals suggests that participants value a balanced approach that provides both informational and emotional appeals. This is consistent with the idea that health messages should incorporate both rational and affective components to maximize persuasive impact (Bekalu et al., 2018). By presenting a mix of factual information about the screening process and relatable narratives of families benefiting from early detection, promotional materials may more effectively engage the target audience.

The positive reception of the screening message, along with participants' expressed interest in its consequences, highlights the importance of framing health communication in terms of both potential benefits and risks. This may reflect the importance of using both gain-framed and loss-framed messages in health promotion materials to effectively encourage screening participation. Gain-framed messages highlight the benefits of engaging in a health behaviour, while loss-framed messages emphasize the costs of not engaging in the behavior (Rothman & Salovey, 1997). The participants' desire to see depictions of both positive and negative screening outcomes reflects this evidence for using mixed framing. Narrative communication theory proposes that audiences may be more receptive to health information conveyed through relatable stories that reduce counterarguing and elicit emotional connections (Bekalu et al., 2018). Collectively, these findings highlight the potential benefits of developing screening promotion materials that include both gain and loss-framed visual narratives to maximize persuasive impact.

In addition, Prospect Theory proposes that individuals are more likely to act when presented with information about the relative advantages and disadvantages of their choices (Rothman & Salovey, 1997). Participants' desire to see both positive outcomes of screening and negative consequences of not participating highlights the potential benefits of using mixed framing in screening promotion materials. This finding is supported by research suggesting that combining gain-framed and loss-framed messages may be most effective for encouraging preventive health behaviors (Jiang et al., 2022).

Participants also emphasized the need for images to provide sufficient detail about the bowel cancer screening process. This finding underscores the importance of using visuals to bridge knowledge gaps and clarify the steps involved in participating. By incorporating images of the test kit, screening procedures, and health care professionals, promotional materials can help alleviate uncertainties and provide a supportive framework for individuals considering screening (Prowse et al., 2024). Yet, we need to highlight the cultural relevance when using pictures of test kits or medical figures, as the study's results are mixed and the qualitative nature of the research does not leave room for generalizability. The trust and credibility associated with medical figures may enhance the persuasive impact of screening messages, as health care provider recommendations have been identified as a strong predictor of cancer screening uptake (Tsipa et al., 2021).

The study also identified discouraging elements that may hinder the effectiveness of screening promotion images. Ambiguous visuals that do not clearly relate to bowel cancer screening, such as images of happy families without contextual cues, may lead to confusion and reduced message comprehension. This finding emphasizes the need for images to have a direct and explicit connection to the intended topic to avoid misinterpretation (Gatting, 2022).

Another discouraging element identified was the presence of items with negative connotations, such as toilets and graphic depictions of organs. These images elicited feelings of disgust and concerns about hygiene, which may discourage individuals from engaging with the screening process. This finding is consistent with research showing that negative emotions, particularly disgust, can act as barriers to health behaviors like cancer screening (Reynolds et al., 2013). The visceral nature of disgust reactions highlights the importance of carefully selecting visual elements to avoid triggering negative emotional responses that may hinder screening participation (Santos et al., 2022).

The current study's findings have important implications for the design of effective bowel cancer screening promotion materials. To maximize the persuasive impact of visual communication, it is recommended that health organizations should incorporate a mix of informational and emotional appeals. To address knowledge gaps and build confidence, materials should provide comprehensive information about the screening process, featuring clear visuals of the test kit, step-by-step procedures, and health care professionals involved. It's crucial that all visual content maintains a direct and obvious connection to bowel cancer screening. When selecting images, special attention must be paid to avoiding content that might provoke negative emotional responses, especially feelings of disgust, as these reactions could deter people from participating in screening programs. This thoughtful approach to visual communication can help create more effective and engaging screening promotion materials that encourage participation while maintaining sensitivity to the audience's emotional responses. The insights gained from this study can inform future studies of discouraging and encouraging messages to different populations and different forms of cancer screening, as well as investigate the advertisements' impact on the potential barriers to participation.

## Limitations and Future Research

The qualitative nature of the research and the relatively small sample size from two specific countries may limit the generalizability of the findings to other populations and cultural contexts. Future research should explore the perceptions of encouraging and discouraging visual elements in bowel cancer screening promotion materials among diverse populations to identify culturally specific preferences and barriers. Additionally, further research is needed to examine the actual behavioral outcomes of exposure to these images. Longitudinal studies that assess the effectiveness of promotional materials incorporating the identified encouraging elements and avoiding discouraging elements on screening uptake rates would provide a more comprehensive understanding of the real-world impact of visual communication strategies. Lastly, some interviews in this study were conducted in Hindi and Bengali and subsequently translated into English for analysis. Despite taking comprehensive measures to ensure accurate and faithful translation, we recognize that some subtle shifts in meaning or nuance may have occurred during the process. Future research may benefit from incorporating back-translation or participant validation to further enhance linguistic fidelity.

## Conclusions

In conclusion, the current study contributes to the growing body of research on effective health communication strategies for promoting bowel cancer screening. The findings highlight the importance of carefully selecting visual elements that resonate with the target audience, provide sufficient information about the screening process, and avoid triggering negative emotions. By applying these insights to the design of screening promotion materials, health organizations can create more persuasive and culturally relevant visual strategies. This can help increase screening uptake, support earlier detection and treatment, and ultimately reduce mortality from bowel cancer.
